# Supplementary material for: Pharmaceutical Evaluation of Levofloxacin Orally Disintegrating Tablet Formulation Using Low Frequency Raman Spectroscopy
Source: Pharmaceutics. 2023 Jul 29;15(8):2041. doi: 10.3390/pharmaceutics15082041 (PMC10459943; doi:10.3390/pharmaceutics15082041)
Supplement: Supplementary file 1 [file pharmaceutics-15-02041-s001.zip › pharmaceutics-2444669-supplementary.pdf]

Supplementary Materials

Pharmaceutical Evaluation of Levofloxacin Orally Disintegrating Tablet Formulation Using Low Frequency Raman Spectroscopy

Yoshihisa Yamamoto [1,*], Mizuho Kajita [2], Yutaro Hirose [2], Naoki Shimada [3], Toshiro Fukami [3,*] and Tatsuo Koide [4]

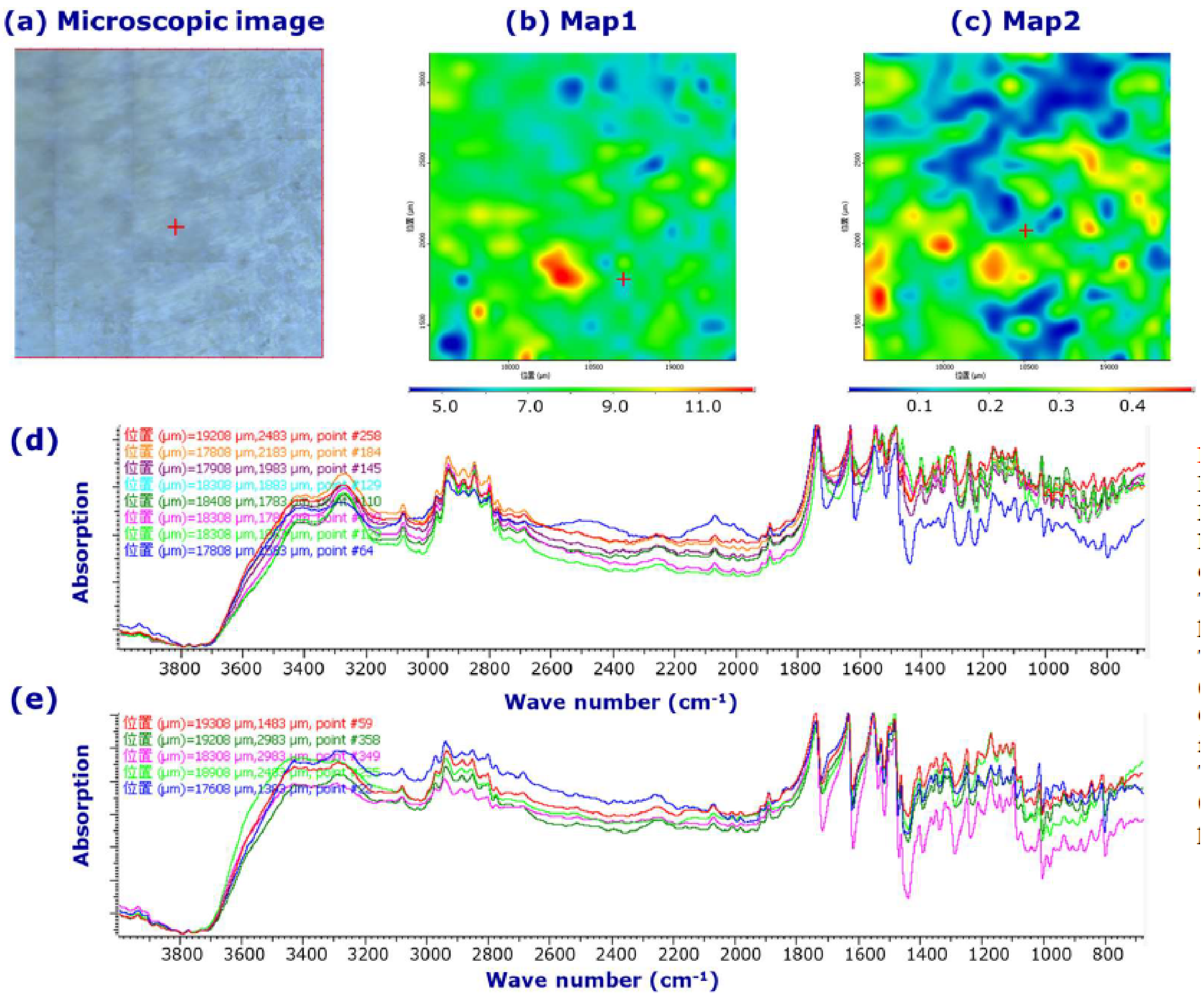

Figure S1. Microscopic and mapping image of LV_{T_CRAVIT} by microscopic IR spectroscopic method (2000 µm × 2000 µm). Microscopic image (a), Map 1; mapping image obtained from the 1700–1800 cm^{-1} peak area ($PA_{1700–1800}$) of each spectrum (Map 1). The red and blue regions indicate high and low peak areas, respectively. The measurement area was 2000 µm × 2000 µm (b), Map 2; mapping image obtained from the correlation to standard microcrystalline cellulose (MCC) spectrum (CR_{MCC}) (Map 2). The red and blue regions indicate high and low correlations, respectively. The measurement area was 2000 µm × 2000 µm (c), IR spectra obtained from the measurement points in the red (d) and blue regions (e) of Map 1.

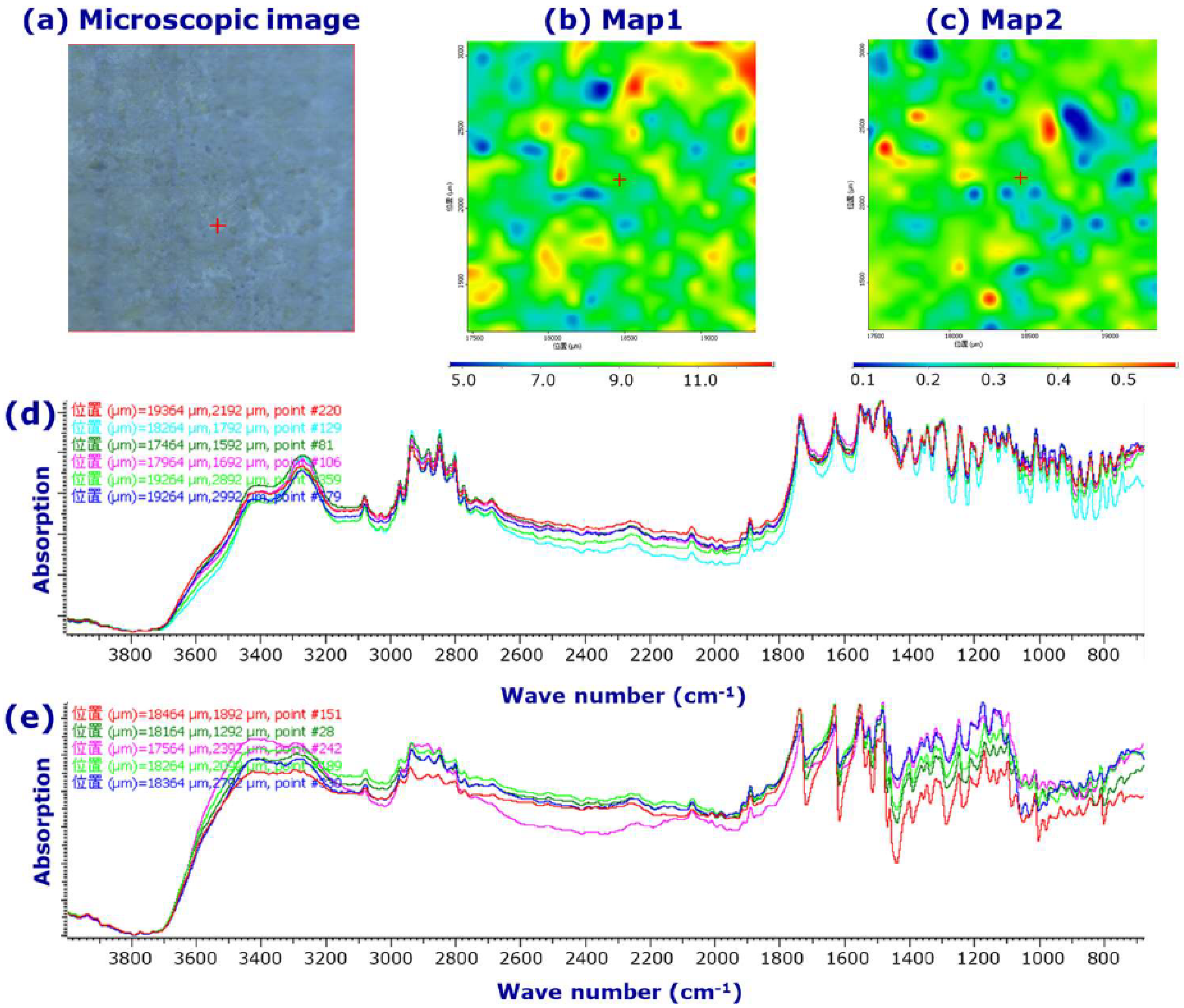

Figure S2. Microscopic and mapping image of LV_{T_NIPRO} by microscopic IR spectroscopic method (2000 μm × 2000 μm). Microscopic image (a), Map 1; mapping image obtained from the 1700–1800 cm^{-1} peak area ($PA_{1700–1800}$) of each spectrum (Map 1). The red and blue regions indicate high and low peak areas, respectively. The measurement area was 2000 μm × 2000 μm (b), Map 2; mapping image obtained from the correlation to standard microcrystalline cellulose (MCC) spectrum (CR_{MCC}) (Map 2). The red and blue regions indicate high and low correlations, respectively. The measurement area was 2000 μm × 2000 μm (c), IR spectra obtained from the measurement points in the red (d) and blue regions (e) of Map 1

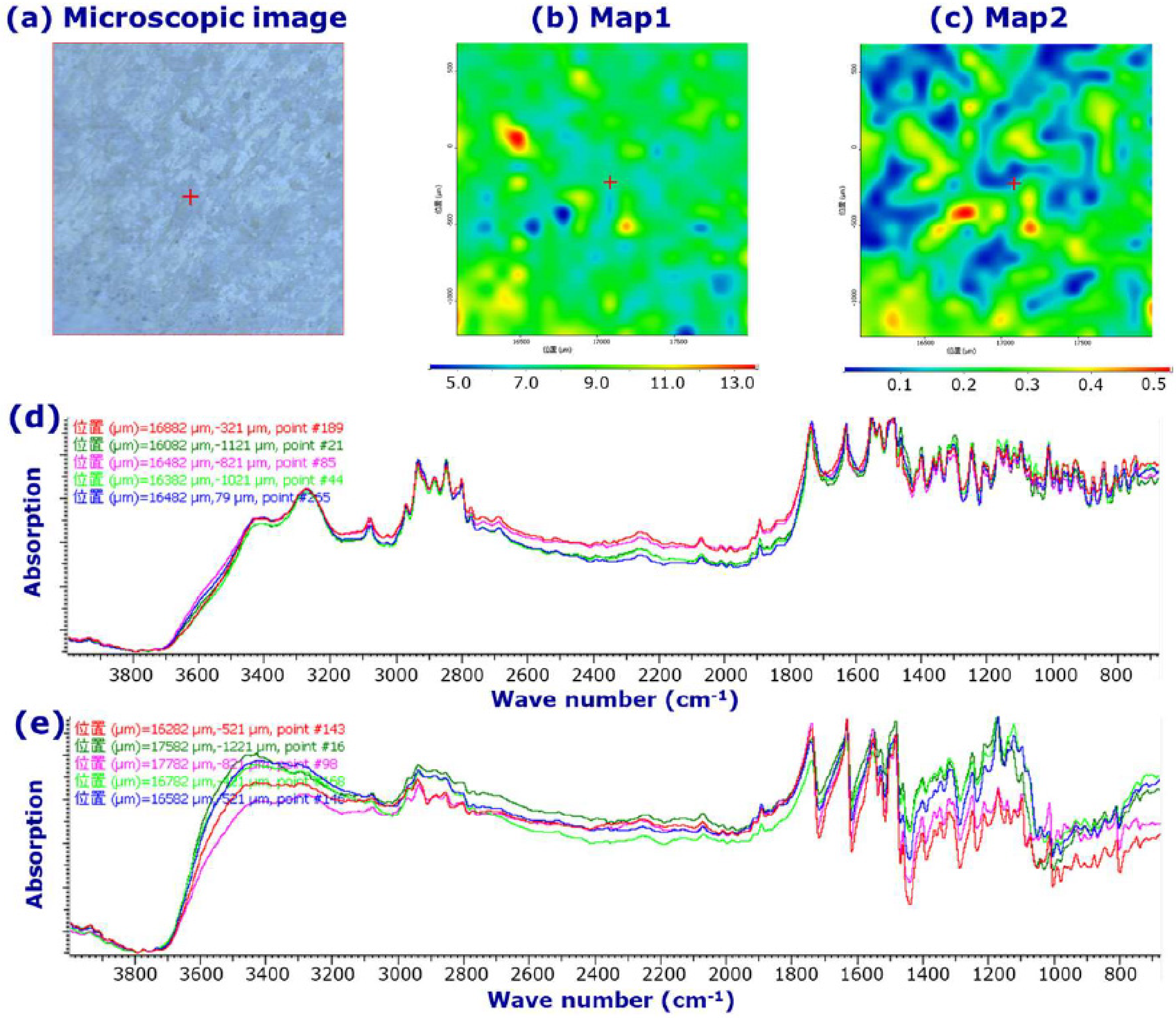

Figure S3. Microscopic and mapping image of LV_{T_SAWAI} by microscopic IR spectroscopic method (2000 μm × 2000 μm). Microscopic image (a), Map 1; mapping image obtained from the 1700–1800 cm^{-1} peak area ($PA_{1700–1800}$) of each spectrum (Map 1). The red and blue regions indicate high and low peak areas, respectively. The measurement area was 2000 μm × 2000 μm (b), Map 2; mapping image obtained from the correlation to standard microcrystalline cellulose (MCC) spectrum (CR_{MCC}) (Map 2). The red and blue regions indicate high and low correlations, respectively. The measurement area was 2000 μm × 2000 μm (c), IR spectra obtained from the measurement points in the red (d) and blue regions (e) of Map 1.

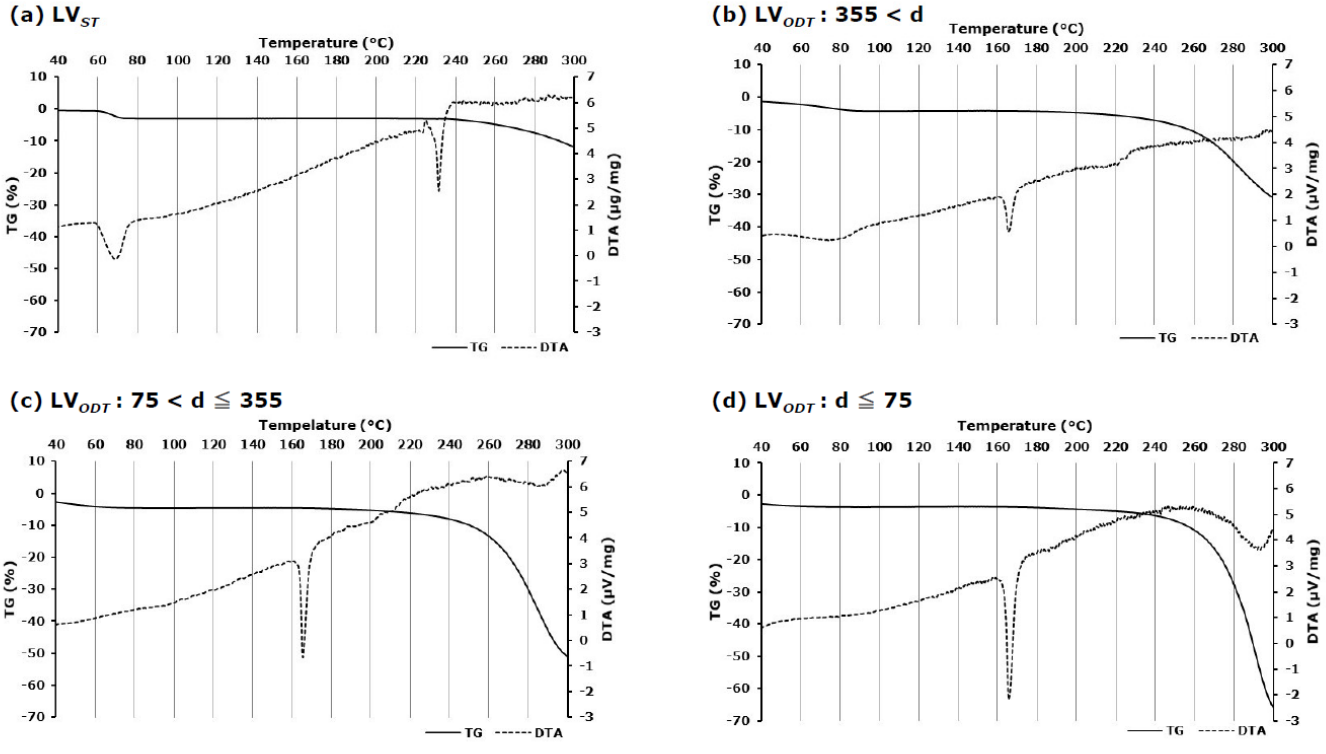

Figure S4. TG-DTA curves of lightly crushed LV$_{ST}$ (a) and LV$_{ODT}$ of several particle size (b–d).

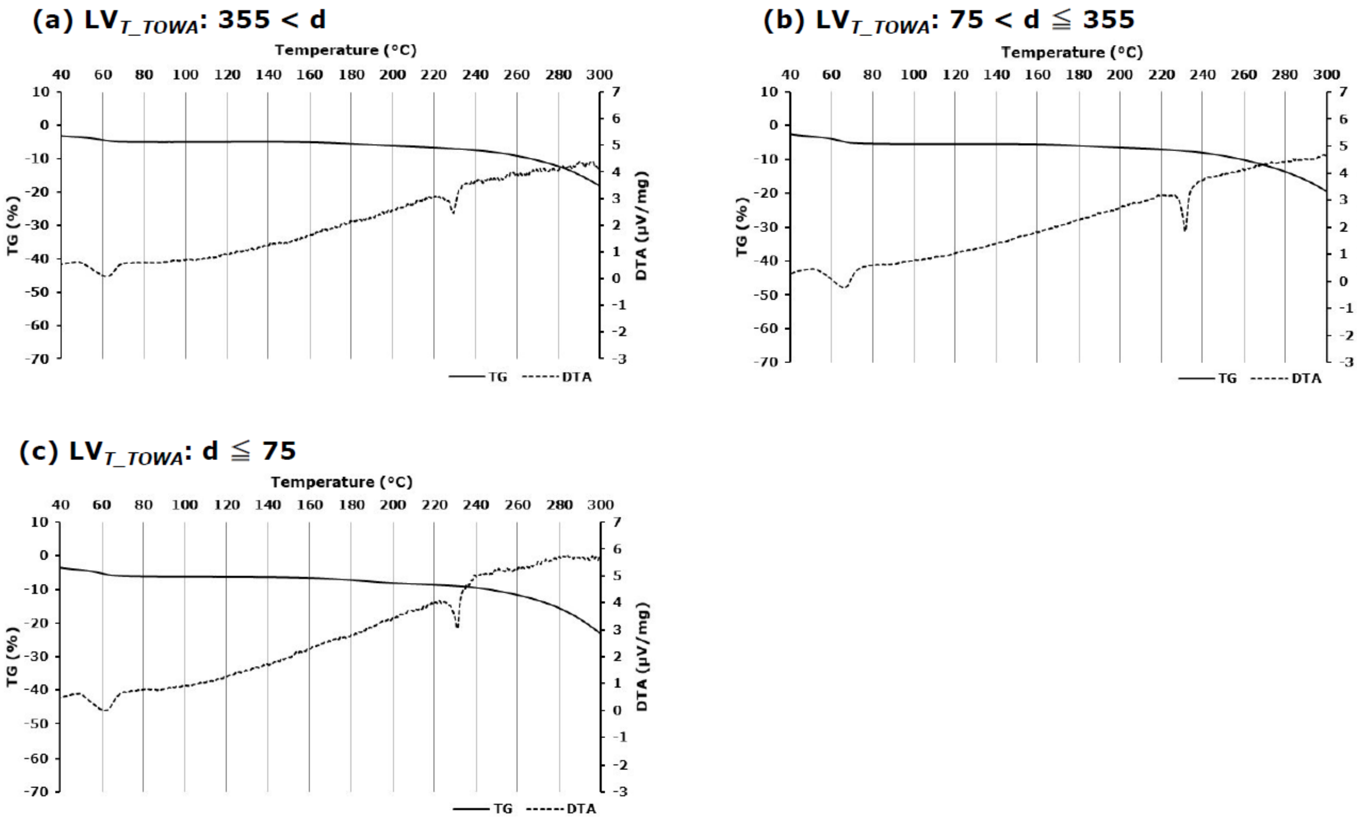

Figure S5. TG-DTA curves of lightly crushed LV$_{T_TOWA}$ of several particle size.

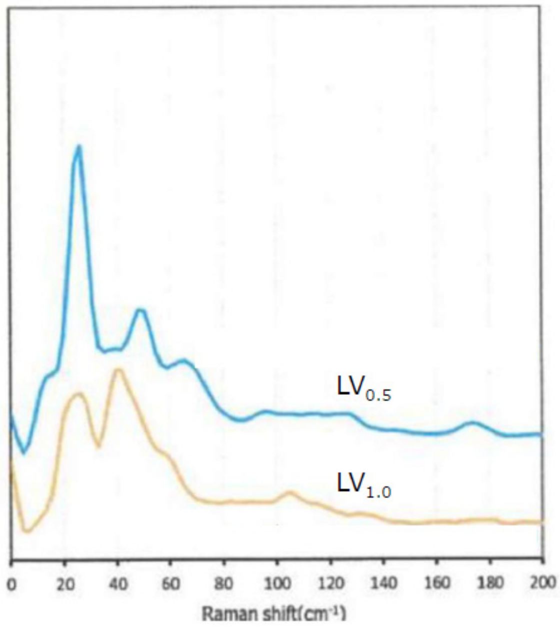

Figure S6. Raman spectrum of experimentally prepared $LV_{0.5}$ and $LV_{1.0}$.

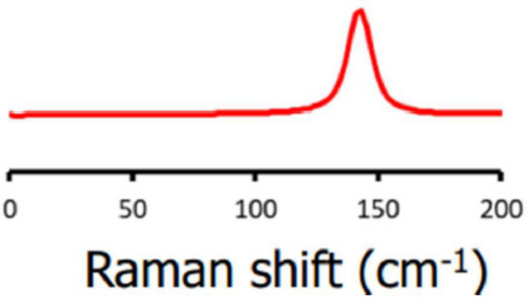

Figure S7. Raman spectra of titanium dioxide.
